# Medical Risk Factors Associated with Cholangiocarcinoma in Taiwan: A Population-Based Case-Control Study

**DOI:** 10.1371/journal.pone.0069981

**Published:** 2013-07-22

**Authors:** Jeffrey S. Chang, Chia-Rung Tsai, Li-Tzong Chen

**Affiliations:** 1 National Institute of Cancer Research, National Health Research Institutes, Tainan, Taiwan; 2 Department of Internal Medicine, National Cheng Kung University Hospital, Tainan, Taiwan; 3 Department of Internal Medicine, Kaohsiung Medical University Hospital, Kaohsiung Medical University, Kaohsiung, Taiwan; 4 Institute of Molecular Medicine, National Cheng Kung University, Tainan, Taiwan; Beijing Institute of Infectious Diseases, China

## Abstract

**Background:**

Cholangiocarcinoma, including intra- and extrahepatic cholangiocarcinoma, is a rare but highly lethal cancer. Despite effort in finding the risk factors of cholangiocarcinoma, the causes of most cholangiocarcinoma remain unknown. This study utilized a population-based case-control design using data from the National Health Insurance Research Database (NHIRD) of Taiwan to assess the medical conditions associated with cholangiocarcinoma.

**Methods:**

5,157 incident cases of cholangiocarcinoma diagnosed during 2004 to 2008 and 20,628 controls matched to the cases on sex, age, and time of diagnosis (reference date for the controls) were identified from the NHIRD. Medical risk factors were ascertained from the NHIRD for each individual. Conditional logistic regression was performed to evaluate the association between cholangiocarcinoma and each medical risk factor.

**Results:**

The results showed that factors associated with an increased risk of cholangiocarcinoma included cholangitis, cholelithiasis, cholecystitis, cirrhosis of liver, alcoholic liver disease, chronic non-alcoholic liver disease, hepatitis B, hepatitis C, diabetes, chronic pancreatitis, inflammatory bowel disease, and peptic ulcer. In addition, sex and age differences were observed.

**Conclusions:**

This study confirms the association between cholangiocarcinoma and several less established risk factors, including diabetes, inflammatory bowel disease, hepatitis B, hepatitis C, and peptic ulcer (proxy for the presence of *Helicobacter Pylori*). Future studies should focus on finding additional environmental and genetic causes of cholangiocarcinoma.

## Introduction

Cholangiocarcinoma, including intra- and extrahepatic cholangiocarcinoma (ICC and ECC), is rare but highly lethal. The established risk factors for cholangiocarcinoma are primary sclerosing cholangitis, liver flukes (*Opistorchis viverrini* and *Clonorchis sinensis*) in endemic regions, cholelithiasis or hepatolithiasis, and congenital biliary tract abnormalities associated with Caroli’s syndrome such as choledochal cysts [1], [2]. Recently, other risk factors have emerged, including viral hepatitis (hepatitis B and hepatitis C), diabetes, and inflammatory bowel disease [1]–[3], but their roles in the pathogenesis of cholangiocarcinoma need to be confirmed, preferably in population-based studies with a large sample size. The goals of this study are: 1) to examine whether the established risk factors of cholangiocarcinoma are also significant for the occurrence of cholangiocarcinoma in Taiwan with a population-based and record-based case-control study using data from the National Health Insurance Research Database (NHIRD) of Taiwan; and 2) to evaluate the significance of various emerging risk factors of cholangiocarcinoma.

## Materials and Methods

This is an analysis of de-identified secondary data; therefore, no informed consent was required. This study was approved by the Research Ethics Committee of the National Health Research Institutes, Taiwan.

### Data Source

Taiwan’s National Health Insurance (NHI) program, run by the Bureau of the National Health Insurance (BNHI), is a single-payer program launched on March 1, 1995 and covers approximately 99% of the 23 million Taiwanese citizens, who have access to inpatient care, ambulatory care, dental care, and prescription drugs from medical facilities contracted with the BNHI. The BNHI routinely monitors the accuracy and the completeness of the claims data of the NHI. The National Health Research Institutes is commissioned by the BNHI to create the NHIRD for medical research using the administrative and health claims data generated by the NHI program.

### Subject Selection

The study subjects were identified from two of the datasets of the NHIRD: 1) Cases were identified from the *Catastrophic Illness Dataset*, a dataset containing health claims data for the treatment of catastrophic illnesses, which consists of thirty categories of diseases that require long-term care, including malignant neoplasms. Case subjects were patients aged 21 or older, newly diagnosed with cholangiocarcinoma [ICC (ICD-9-CM code: 155.1) and ECC (ICD-9-CM code: 156.1)] between January 1, 2004 and December 31, 2008. To reduce the financial hardship associated with catastrophic illnesses, patients with a catastrophic illness, including cancer, are issued a *certificate of catastrophic illness* to be exempted from all co-payments for catastrophic illness treatments. To receive a *certificate of catastrophic illness*, one must have an official certificate of diagnosis issued by the hospital with support from pathology and/or imagery studies (*e.g.* conventional X-ray, CT, ultrasound) or laboratory tests. Thus, the accuracy of cancer cases identified from the *Catastrophic Illness Dataset* should be high; 2) Control subjects were identified from the *Longitudinal Health Insurance Database 2005*, a database containing the claims data of one million people randomly sampled from the 2005 NHIRD enrollment file. This random sample is representative of the entire insured population of Taiwan. For each cholangiocarcinoma case, 4 cancer-free controls individually matched to the case on sex, age (birthday), and the time of case diagnosis (reference date for the controls) were identified.

### Assessment of Risk Factors

The medical risk factors included in this study were those that have been previously shown to be associated with or possibly related to the development of cholangiocarcinoma. They included: 1) **biliary tract diseases:** choledochal cysts (ICD-9-CM code: 751.69), cholangitis (ICD-9-CM code: 576.1), cholelithiasis (ICD-9-CM code: 574), cholecystitis (ICD-9-CM codes: 575.0 and 575.1), and liver flukes (ICD-9-CM codes: 121.0, 121.1, and 121.3); 2) **chronic liver diseases:** hemochromatosis (ICD-9-CM code: 275.0), cirrhosis of liver (non-alcohol-related) (ICD-9-CM codes: 571.5 and 571.6), alcoholic liver disease (alcoholic cirrhosis included) (ICD-9-CM codes: 571.0, 571.1, 571.2, and 571.3), chronic non-alcoholic liver disease (ICD-9-CM code: 571.8), hepatitis B (ICD-9-CM codes: 070.2, 070.3, and V02.61), and hepatitis C (ICD-9-CM codes: 070.41, 070.44, 070.51, 070.54, V02.62, and 070.7); 3) **endocrine diseases**: diabetes (ICD-9-CM code: 250); and 4) **digestive diseases:** chronic pancreatitis (ICD-9-CM code: 577.1), inflammatory bowel disease (ICD-9-CM codes: 556, 557.0, and 555), and peptic ulcer (ICD-9-CM codes: 531, 532, and 533). To avoid diagnostic bias, meaning that cholangiocarcinoma case subjects might have been more likely to be diagnosed with other medical conditions while visiting hospitals for symptoms associated with the development of cholangiocarcinoma, or reverse causality, risk factors occurring during the year prior to the diagnosis of cholangiocarcinoma (reference date for the matched controls) were excluded. In addition, we excluded medical risk factors that were treated after the diagnosis of cholangiocarcinoma, because of the possibility that those medical risk factors may have occurred after the development of cholangiocarcinoma and may thus confound the temporality of events.

### Statistical Analysis

Conditional logistic regression was performed to calculate the odds ratio (OR) and 95% confidence interval (CI) to evaluate the association between ICC or ECC and each medical risk factor with age, sex, and the time of diagnosis (reference date for the controls) as matching variables. Many of the medical risk factors included in the analysis may represent the same disease process with some risk factors potentially being the intermediate factors for the others. For example, cholelithiasis may lead to inflammation of the bile duct (cholangitis), resulting in an increased risk of cholangiocarcinoma. To assess the independent association between a medical risk factor and cholangiocarcinoma, multivariable analysis was performed to adjust for possible intermediate factors [4]. Additional exploratory analyses were performed to compare the association between each risk factor and cholangiocarcinoma stratified by sex and age (≦65 years old vs. >65 years old). Statistical tests of heterogeneity were performed by comparing the full model containing the product term (risk factor × sex or risk factor × age) to the submodel without the product term using the log-likelihood ratio test.

## Results

A total of 5,157 cases of cholangiocarcinoma (2,978 cases of ICC and 2,179 cases of ECC) were identified from the NHIRD for the defined period of interest. For every case, four individually age (birthday)-, sex-, and time- matched controls were identified for a total of 20,628 controls (11,912 controls for ICC and 8,716 controls for ECC). Among the ICC cases and the matched controls, 56% were male and 67% were greater than 60 years old. Among the ECC cases and the matched controls, 54% were male and 70% were greater than 60 years old (Table 1).

**Table 1:** Distributions of age and sex of the study subjects.

Characteristics	Intrahepatic cholangiocarcinoma		Extrahepatic cholangiocarcinoma	
	Case	Control	Case	Control
	N = 2,978	N = 11,912	N = 2,179	N = 8,716
	n (%)	n (%)	n (%)	n (%)
**Age (year)**				
21–40	87 (2.9)	348 (2.9)	45 (2.1)	180 (2.1)
41–50	289 (9.7)	1,156 (9.7)	183 (8.4)	732 (8.4)
51–60	598 (20.1)	2,392 (20.1)	414 (19.0)	1,656 (19.0)
61–70	864 (29.0)	3,456 (29.0)	582 (26.7)	2,328 (26.7)
71–80	792 (26.6)	3,168 (26.6)	627 (28.8)	2,508 (28.8)
≧81	348 (11.7)	1,392 (11.7)	328 (15.0)	1,312 (15.0)
**Sex**				
Male	1,670 (56.1)	6,680 (56.1)	1,184 (54.3)	4,736 (54.3)
Female	1,308 (43.9)	5,232 (43.9)	995 (45.7)	3,980 (45.7)

### Biliary Tract Diseases

Choledochal cysts (ICC: OR = 20.0, 95% CI: 4.4–91.3; ECC: OR = 20.0, 95% CI: 2.3–171.2), cholangitis (ICC: OR = 27.8, 95% CI: 20.2–38.4; ECC: OR = 12.6, 95% CI: 9.4–16.9), cholelithiasis (see Table 2 for ORs by locations of the stones), and cholecystitis (ICC: OR = 6.3, 95% CI: 4.9–8.1; ECC: OR = 4.7, 95% CI: 3.5–6.3) were all significantly (p<0.05) associated with an increased risk of cholangiocarcinoma (Table 2). The number of subjects with liver flukes was too rare for analysis.

**Table 2:** The association between medical risk factors and cholangiocarcinoma by disease sites.

	Intrahepatic cholangiocarcinoma				Extrahepatic cholangiocarcinoma			
Risk factors	Case	Control	OR^a^ (95% CI)	P-value^a^	Case	Control	OR^a^ (95% CI)	P-value^a^
	N = 2,978	N = 11,912			N = 2,179	N = 8,716		
	n (%)	n (%)			n (%)	n (%)		
**Biliary tract diseases**								
Choledochal cyst	10 (0.3)	2 (0.02)	20.0 (4.4–91.3)	0.0001	5 (0.2)	1 (0.01)	20.0 (2.3–171.2)	0.006
Cholangitis	304 (10.2)	51 (0.4)	27.8 (20.2–38.4)	<0.0001	188 (8.6)	63 (0.7)	12.6 (9.4–16.9)	<0.0001
Cholelithiasis								
Gallbladder+bile duct	258 (8.7)	61 (0.5)	21.2 (15.8–28.3)	<0.0001	145 (6.7)	58 (0.7)	11.8 (8.6–16.3)	<0.0001
Gallbladder only	254 (8.5)	394 (3.3)	3.2 (2.7–3.8)	<0.0001	173 (7.9)	311 (3.6)	2.6 (2.1–3.2)	<0.0001
Bile duct only	147 (4.9)	35 (0.3)	21.1 (14.4–30.9)	<0.0001	99 (4.5)	33 (0.4)	13.7 (9.2–20.4)	<0.0001
Unspecified location	34 (1.1)	38 (0.3)	4.5 (2.8–7.4)	<0.0001	19 (0.9)	37 (0.4)	2.8 (1.6–5.0)	0.0004
Cholecystitis	157 (5.3)	103 (0.9)	6.3 (4.9–8.1)	<0.0001	93 (4.3)	83 (1.0)	4.7 (3.5–6.3)	<0.0001
Liver Flukes	2 (0.07)	1 (0.01)	8.0 (0.7–88.2)	0.09	1 (0.05)	0 (0.0)	–	–
**Chronic liver diseases**								
Hemochromatosis	0 (0.0)	1 (0.01)	–	–	0 (0.0)	2 (0.02)	–	–
Cirrhosis of liver	303 (10.2)	160 (1.3)	8.0 (6.6–9.8)	<0.0001	104 (4.8)	112 (1.3)	3.9 (3.0–5.1)	<0.0001
Alcoholic liver disease	110 (3.7)	121 (1.0)	3.8 (2.9–5.0)	<0.0001	46 (2.1)	74 (0.9)	2.5 (1.7–3.6)	<0.0001
Chronic non-alcoholic liver disease	156 (5.2)	236 (2.0)	2.7 (2.2–3.3)	<0.0001	89 (4.1)	174 (2.0)	2.1 (1.6–2.7)	<0.0001
Hepatitis B	257 (8.6)	229 (1.9)	4.9 (4.1–5.9)	<0.0001	119 (5.5)	164 (1.9)	3.0 (2.4–3.8)	<0.0001
Hepatitis C	193 (6.5)	138 (1.2)	5.8 (4.7–7.3)	<0.0001	57 (2.6)	102(1.2)	2.3 (1.6–3.1)	<0.0001
**Endocrine diseases**								
Diabetes	932 (31.3)	2,296 (19.3)	2.0 (1.8–2.2)	<0.0001	661 (30.3)	1,731 (19.9)	1.8 (1.6–2.0)	<0.0001
**Digestive diseases**								
Chronic pancreatitis	37 (1.2)	22 (0.2)	7.0 (4.1–11.9)	<0.0001	17 (0.8)	18 (0.2)	3.8 (1.9–7.3)	<0.0001
Inflammatory bowel disease	165 (5.5)	336 (2.8)	2.0 (1.7–2.4)	<0.0001	88 (4.0)	218(2.5)	1.6 (1.3–2.1)	0.0001
Peptic ulcer	1,455 (48.9)	3,176 (26.7)	2.7 (2.5–2.9)	<0.0001	983 (45.1)	2,358 (27.1)	2.3 (2.0–2.5)	<0.0001

a Oa. dds ratio (OR), 95% confidence interval (CI), and p-value were derived using conditional logistic regression with age, sex, and the time of diagnosis (reference date for the controls) as matching variables.

### Chronic Liver Diseases

Cirrhosis of the liver (ICC: OR = 8.0, 95% CI: 6.6–9.8; ECC: OR = 3.9, 95% CI: 3.0–5.1), alcoholic liver disease (ICC: OR = 3.8, 95% CI: 2.9–5.0; ECC: OR = 2.5, 95% CI: 1.7–3.6), chronic non-alcoholic liver disease (ICC: OR = 2.7, 95% CI: 2.2–3.3; ECC: OR = 2.1, 95% CI: 1.6–2.7), hepatitis B (ICC: OR = 4.9, 95% CI: 4.1–5.9; ECC: OR = 3.0, 95% CI: 2.4–3.8), and hepatitis C (ICC: OR = 5.8, 95% CI: 4.7–7.3; ECC: OR = 2.3, 95% CI: 1.6–3.1) were all associated with an elevated risk of cholangiocarcinoma (Table 2). Hemochromatosis did not have a sufficient number for analysis.

### Endocrine Diseases

Diabetes was associated with an increased risk for both ICC (OR = 2.0, 95% CI: 1.8–2.2) and ECC (OR = 1.8, 95% CI: 1.6–2.0) (Table 2).

### Digestive Diseases

Chronic pancreatitis (ICC: OR = 7.0, 95% CI: 4.1–11.9; ECC: OR = 3.8, 95% CI: 1.9–7.3), inflammatory bowel disease (ICC: OR = 2.0, 95% CI: 1.7–2.4; ECC: OR = 1.6, 95% CI: 1.3–2.1), and peptic ulcer (ICC: OR = 2.7, 95% CI: 2.5–2.9; ECC: OR = 2.3, 95% CI: 2.0–2.5) were all associated with an increased risk of cholangiocarcinoma (Table 2).

### Multivariable Analysis Adjusted for Possible Intermediate Factors (Table 3)

Adjusting for possible intermediate factors showed varying degrees of impact on different risk factors. For some (chronic non-alcoholic liver disease, diabetes, and inflammatory bowel disease), adjusting for intermediate factors changed the ORs by 10% or less, which suggests a major influence from alternative mechanisms rather than the intermediate factors examined. The association between other risk factors (cholelithiasis, hepatitis B, and hepatitis C) and cholangiocarcinoma can be partly accounted by the examined intermediate risk factors (>10% change in ORs), although alternative mechanisms remained a possibility, since the ORs, although much weakened, still remained significant after adjusting for intermediate factors. For chronic pancreatitis, the association with cholangiocarcinoma can be largely explained by cholelithiasis, cholangitis, and cirrhosis of liver, with the association between chronic pancreatitis and ECC becoming null after adjusting for the three intermediate factors.

**Table 3:** Multivariable analysis adjusted for possible intermediate factors for the association between selected medical risk factors and cholangiocarcinoma by disease sites.

		Intrahepatic cholangiocarcinoma		Extrahepatic cholangiocarcinoma	
Risk factors	Possible intermediate factors	OR unadjusted^a^(95% CI)	OR adjusted^b^(95% CI)	OR unadjusted^a^(95% CI)	OR adjusted^b^(95% CI)
Cholelithiasis	Cholangitis	6.6 (5.8–7.5)	4.3 (3.7–4.9)	4.7 (4.1–5.4)	3.3 (2.8–3.9)
Chronic non-alcoholic liver disease	Cirrhosis of liver	2.7 (2.2–3.3)	2.4 (1.9–2.9)	2.1 (1.6–2.7)	2.0 (1.5–2.6)
Hepatitis B	Cirrhosis of liver	4.9 (4.1–5.9)	3.5 (2.9–4.3)	3.0 (2.4–3.8)	2.6 (2.0–3.4)
Hepatitis C	Cirrhosis of liver	5.8 (4.7–7.3)	3.5 (2.7–4.4)	2.3 (1.6–3.1)	1.8 (1.3–2.5)
Diabetes	Cholelithiasis and cholangitis	2.0 (1.8–2.2)	1.8 (1.6–2.0)	1.8 (1.6–2.0)	1.7 (1.5–1.9)
Chronic pancreatitis	Cholelithiasis, cholangitis, and cirrhosis of liver	7.0 (4.1–11.9)	2.5 (1.3–4.7)	3.8 (1.9–7.3)	0.8 (0.4–2.0)
Inflammatory bowel disease	Cholelithiasis and cholangitis	2.0 (1.7–2.4)	1.7 (1.4–2.1)	1.6 (1.3–2.1)	1.5 (1.1–1.9)

a Odds ratio (OR) and 95% confidence interval (CI) were derived using conditional logistic regression with age, sex, and the time of diagnosis (reference date for the controls) as matching variables.

b Odds ratio (OR) and 95% confidence interval (CI) were derived using conditional logistic regression with age, sex, and the time of diagnosis (reference date for the controls) as matching variables and adjusted for possible intermediate factors.

### Results Stratified by Sex and Age (Table 4)

Cholelithiasis was associated with a stronger increased risk of ICC (male: OR = 4.7, 95% CI: 3.9–5.6; female: OR = 9.3, 95% CI: 7.7–11.2; interaction p-value =  <0.0001) and ECC (male: OR = 3.8, 95% CI: 3.1–4.7; female: OR = 5.8, 95% CI: 4.7–7.1; interaction p-value = 0.005) among females than among males. Peptic ulcer was more strongly associated with an increased risk of ICC among females (OR = 3.1, 95% CI: 2.7–3.5) than among males (OR = 2.4, 95% CI: 2.2–2.7) (interaction p-value = 0.006). Cholangitis was more strongly associated with an increased risk of ECC among females (OR = 22.0, 95% CI: 13.6–35.4) than among males (OR = 7.8, 95% CI: 5.3–11.5) (interaction p-value = 0.001). Both hepatitis B (male: OR = 4.3, 95% CI: 3.2–5.9; female: OR = 1.7, 95% CI: 1.1–2.5; interaction p-value = 0.0003) and hepatitis C (male: OR = 3.0, 95% CI: 2.0–4.7; female: OR = 1.5, 95% CI: 0.9–2.6; interaction p-value = 0.05) were associated with a stronger increased risk of ECC in males than in females. Diabetes was associated with a stronger increased risk of ECC in females (OR = 2.1, 95% CI: 1.8–2.5) than in males (OR = 1.6; 95% CI: 1.4–1.8) (interaction p-value = 0.01).

**Table 4:** Results of the association between medical risk factors and cholangiocarcinoma stratified by sex and age, statistically significant interaction only (p<0.05).

	Intrahepatic cholangiocarcinoma			Extrahepatic cholangiocarcinoma		
Risk factors	OR (95% CI)^a^		Interaction P-value^a^	OR^a^ (95% CI)^a^		Interaction P-value^a^
	Sex					
	Male	Female		Male	Female	
Cholangitis				7.8 (5.3–11.5)	22.0 (13.6–35.4)	0.001
Cholelithiasis	4.7 (3.9–5.6)	9.3 (7.7–11.2)	<0.0001	3.8 (3.1–4.7)	5.8 (4.7–7.1)	0.005
Hepatitis B				4.3 (3.2–5.9)	1.7 (1.1–2.5)	0.0003
Hepatitis C				3.0 (2.0–4.7)	1.5 (0.9–2.6)	0.05
Diabetes				1.6 (1.4–1.8)	2.1 (1.8–2.5)	0.01
Peptic ulcer	2.4 (2.2–2.7)	3.1 (2.7–3.5)	0.006			
	**Age**					
	**≦65**	**>65**		**≦65**	**>65**	
Cholelithiasis				5.7 (4.5–7.4)	4.2 (3.5–5.0)	0.05
Cirrhosis of liver	11.5 (8.4–15.7)	6.0 (4.6–7.8)	0.002	6.4 (4.0–10.2)	2.9 (2.1–4.1)	0.01

a Odds ratio (OR), 95% confidence interval (CI), and interaction p-values were derived using conditional logistic regression with age, sex, and the time of diagnosis (reference date for the controls) as matching variables.

The association between cholelithiasis and an increased risk of ECC was stronger in those aged 65 years or younger (OR = 5.7; 95% CI: 4.5–7.4) compared to those greater than 65 years old (OR = 4.2; 95% CI: 3.5–5.0) (interaction p-value = 0.05). Cirrhosis of liver was associated with an increased risk of ICC (≦65 years old: OR = 11.5, 95% CI: 8.4–15.7; >65 years old: OR = 6.0, 95% CI: 4.6–7.8; interaction p-value = 0.002) and ECC (≦65 years old: OR = 6.4, 95% CI: 4.0–10.2; >65 years old: OR = 2.9, 95% CI: 2.1–4.1; interaction p-value = 0.01) more strongly for subjects aged 65 years or younger.

## Discussion

In the current analysis, an increased risk of cholangiocarcinoma was observed for cholangitis, cholelithiasis, cholecystitis, cirrhosis of liver, alcoholic liver disease, chronic non-alcoholic liver disease, hepatitis B, hepatitis C, diabetes, chronic pancreatitis, inflammatory bowel disease, and peptic ulcer. Sex and age differences were observed for the association between several medical conditions and cholangiocarcinoma.

Cholangitis was positively associated with cholangiocarcinoma in our analysis, consistent with results from previous studies [3], [5]. The cholangitis identified from the NHIRD likely included some cases of primary sclerosing cholangitis, which does not have a separate ICD-9 code. Various cohort studies showed an increased occurrence of cholangiocarcinoma in patients with primary sclerosing cholangitis, ranging from 10% to 13% [6]–[8].

Our result showing an elevated risk of cholangiocarcinoma associated with cholelithiasis concurs with results from previous publications [3], [5], [9]–[11]. The oncogenic process may result from chronic inflammation around the area of the bile duct harboring the stone. Since there is not an ICD-9 code specific for hepatolithiasis, it is likely that a proportion of our study subjects with cholelithiasis actually had hepatolithiasis. A previous nationwide study indicated that approximately 20% of biliary stones diseases in Taiwan were hepatolithiasis [12], which has been associated with an elevated risk of ICC [13], [14].

The current analysis showed an increased cholangiocarcinoma risk associated with hepatitis B or C. Among the 12 previously published studies examining the relationship between hepatitis B and ICC, 8 showed a statistically significant positive association [14]–[21], three showed a non-significantly positive association [13], [22], [23], and one reported a null association [10]. A positive association between hepatitis C and ICC has been reported by 8 out of 11 studies [3], [10], [13]–[17], [20], [22]–[24]. Among the four studies examining hepatitis B and ECC risk, one had too few subjects with hepatitis B infection to produce a meaningful result [17], one reported a positive association [25], and two reported a non-significantly elevated risk [26], [27]. Of the three studies on the association between hepatitis C and ECC risk, two reported a non-significant positive association [17], [24] and one observed a null association [25]. Overall, the positive association between ICC and hepatitis B or C is consistent across studies, which is supported by two recent meta-analyses (for hepatitis B and ICC: meta-RR estimated by Li et al.  = 3.42, 95% CI: 2.46–43.74 [28] and meta-RR estimated by Palmer et al.  = 5.54, 95% CI: 3.19–9.63 [29]; for hepatitis C and ICC: meta-OR  = 4.84, 95% CI: 2.41–9.71 [29]) The association between ECC and hepatitis B or C needs further investigation. The meta-analysis by Li et al. indicated that hepatitis B may increase the risk of ECC, but the result was not statistically significant (meta-OR  = 1.46, 95% CI: 0.98–2.17) [28]. Hepatocytes and cholangiocytes develop from the same progenitor cells [30]; therefore, the same oncogenic process induced by hepatitis B and hepatitis C in the development of hepatocellular carcinoma may also induce the occurrence of cholangiocarcinoma.

Cirrhosis of liver was more common among cholangiocarcinoma cases than controls and this has also been reported by another study [3]. Cirrhosis may result from a wide array of risk factors including hepatitis B or C infection. Cirrhosis represents a state of chronic liver inflammation and damage, which may be accompanied by malignant changes of the bile ducts.

The positive association between alcoholic liver disease and cholangiocarcinoma in our analysis is consistent with results from previous studies [3], [5], [10]. As alcoholic liver disease is the result of excessive alcohol drinking, it suggests that alcohol consumption may be a risk factor for cholangiocarcinoma [2]. We could not assess the association between alcohol and cholangiocarcinoma due to the lack of alcohol consumption data in the NHIRD.

Our observation of the positive association between diabetes and cholangiocarcinoma is consistent with some of the previous studies [3], [9], [10], [14], [26], [31], though other studies did not observe such association [5], [17], [32]. A recent case-control study with 612 ICC cases and 594 controls showed that diabetes was associated a 3.6 fold increase in the risk of ICC [33]. Furthermore, compared to diabetic patients not treated with metformin, an anti-diabetic drug, diabetic patients who received metformin treatment had a lower risk of ICC (OR = 0.4, 95% CI: 0.2–0.9), which further strengthened the association between diabetes and ICC [33]. Diabetes has been linked to an increased risk of biliary stones [34], one of the possible mediators in the association between diabetes and cholangiocarcinoma. However, our analysis showed an association between diabetes and cholangiocarcinoma even after accounting for the influence of biliary stones, suggesting the existence of an alternative mechanism. Whether the relationship between diabetes and cholangiocarcinoma is direct or through other intermediate risk factors such as obesity remains to be evaluated [9], [35]–[37]. Obesity could increase the risk of cholangiocarcinoma by affecting the levels of leptin, adiponectin, and pro-inflammatory cytokines [37]. Due to the lack of weight and height data in the NHIRD, we were not able to disentangle the relationship between diabetes, obesity, and cholangiocarcinoma.

Peptic ulcer was more prevalent among cholangiocarcinoma cases than controls, consistent with the result from the only other study that examined such association [3]. This suggests that *Helicobacter Pylori* (*H. Pylori*), a major risk factor for peptic ulcer [38], may play a role in the development of cholangiocarcinoma. Bulajic *et* *al.* reported a high correlation between the presence of *H. Pylori* in the stomach and in the bile, and *H. Pylori* in the bile was associated with cholangiocarcinoma [39]. A meta-analysis of 10 case-control studies, which included 205 hepatobiliary tract cancer cases and 263 controls, reported an increased risk of hepatobiliary tract cancer associated with *Helicobacter* species [40]. However, further investigations are required to establish a causal relationship between *H. Pylori* and cholangiocarcinoma.

An increased risk of cholangiocarcinoma associated with inflammatory bowel disease in the current study has also been previously reported [3]. Inflammatory bowel disease has extraintestinal manifestations, including primary sclerosing cholangitis [41] and cholelithiasis [42], two known risk factors for cholangiocarcinoma [43]. However, our analysis showed that primary sclerosing cholangitis (included on the cholangitis variable due to the lack of a separate ICD-9-CM code for primary sclerosing cholangitis) and cholelithiasis do not account for all of the cholangiocarcinoma predisposing effect of inflammatory bowel disease, suggesting the roles of additional factors.

The current analysis showed a positive association between chronic pancreatitis and cholangiocarcinoma. Approximately 3–23% of patients with chronic pancreatitis develop biliary stricture, which may lead to conditions associated with an increased risk of cholangiocarcinoma, including cholangitis, biliary cirrhosis, and cholelithiasis [44]. Our analyses showed that cholangitis, biliary cirrhosis, and cholelithiasis may explain most of the association between chronic pancreatitis and cholangiocarcinoma.

The associations between several medical conditions and cholangiocarcinoma differed by sex in our study. For example, cholelithiasis was associated with a stronger increased risk of cholangiocarcinoma among females than among males. Sex is a strong risk factor associated with gallstone formation and estrogen is thought to be a contributing factor [45]. Variations in the estrogen receptor genes were associated with risk of cholangiocarcinoma, supporting the role of estrogen [46]. Sex hormones may also contribute to the gender difference in the association between hepatitis B or C and ECC. Levels of plasma testosterone and polymorphisms of genes on the androgen signaling pathway have been shown to influence the risk of hepatocellular carcinoma among male hepatitis B virus carriers [47]. Similar mechanism may operate in the association between viral hepatitis and ECC. Alternatively, since men are known to drink more than women in Taiwan [48], the synergistic interaction between viral hepatitis and alcohol consumption may contribute to the higher risk of ECC among men.

Several positive associations were stronger among younger subjects (≦65 years old vs. >65 years old), including the association between cirrhosis of liver and ICC or ECC and between cholelithiasis and ECC. Though a multitude of explanations may explain these differences by age, it is possible that those who develop cholangiocarcinoma at a younger age may carry the susceptible genetic variations. Genetic variations conferring differential risk of cholangiocarcinoma have been reported [46], [49].

The current study has several limitations. The NHIRD only contains medical claims data and does not include information on potential confounders, including annual household income, education, and lifestyle factors such as smoking and alcohol consumption. Although we tried to reduce the possibility of reverse causality by excluding medical risk factors diagnosed within one year before the case’s diagnosis of cholangiocarcinoma, the existence of reverse causality cannot be completely ruled out. Since the medical risk factors assessed by this study were mostly chronic conditions, it was difficult to determine the age of initial diagnosis for those medical risk factors using our database. The age of diagnosis could be any time between birth up to one year before the diagnosis of cholangiocarcinoma (reference date for the controls). For that reason, we were not able to further evaluate the relationship between cholangiocarcinoma and medical risk factors diagnosed at different age periods (e.g. during childhood vs. adulthood). Another limitation is that the NHIRD only records medical claims data and does not indicate whether a person is cured of a medical condition; therefore, our analysis could not distinguish those who were cured from those who were not for condition such as peptic ulcer. However, this limitation should have affected our analysis minimally since most of the medical conditions assessed in our analysis are chronic diseases that require long-term treatment. The major strength of this study is its population-based and record-based nature, which ensures that the results generated from this study is minimally affected by selection and recall biases commonly associated with case-control studies. In addition, the current analysis has a large sample size, which provides the study with high statistical power and precision.

Overall, our study supports the association between cholangiocarcinoma and medical conditions previously reported to increase cholangiocarcinoma risk, including several less established risk factors such as diabetes, inflammatory bowel disease, hepatitis B, hepatitis C, and peptic ulcer (proxy for the presence of *H. Pylori*). Future studies should focus on finding additional environmental and genetic causes of cholangiocarcinoma, because the causes for the majority of cholangiocarcinoma remain unknown [43].
